# 924 Pre-Mixed Vasopressor Transition: A Burn Unit ICU Case Study That Led to Hospital Change

**DOI:** 10.1093/jbcr/iraf019.455

**Published:** 2025-04-01

**Authors:** Kimberly Siemons, Ashley Hoague, Mary McCoy, Toral Patel, Michael Smock, Shari Wojewoda

**Affiliations:** Mercy Hospital St. Louis Burn Unit; Mercy Hospital St. Louis Burn Unit; Mercy Hospital St. Louis Burn Unit; Mercy Hospital St. Louis Burn Unit; Mercy Hospital St. Louis Burn Unit; Mercy Hospital St. Louis Burn Unit

## Abstract

**Introduction:**

When patients are critically ill, our hospital practice has been to have pharmacy mix the vasopressor medication infusion bags. The provider enters the order, pharmacy prepares the medication, then the nurse hangs and programs the medication into the pump for delivery to the patient. Nurses must be diligent to reorder the medication in a timely manner to ensure these vasopressor infusion bags do not run empty. Many Intensive Care Unit (ICU) nurses are comfortable mixing Norepinephrine infusion bags when needed due to the critical needs of patients on their units. However, many ICU nurses are not comfortable mixing Epinephrine medication infusions when needed. This lack of comfort and knowledge along with the delivery process led to an unfortunate outcome in this case scenario review.

**Methods:**

Case Study: A 69-year-old patient was admitted to the hospital for lower leg and back pain, cellulitis, red macules, bruising and possible Stevens Johnson Syndrome. An Epinephrine infusion was ordered and hung at 1004. The Epinephrine infusion was started at a rate of 206.4 mL/HR (0.8 mcg/kg/min) from a 250 mL bag. The infusion was titrated up several times over the next 50 minutes with a final infusion rate of 258 mL/HR (1 mcg/kg/min). At 1108 the Epinephrine replacement bag was reordered from pharmacy. The Epinephrine infusion ultimately ran empty at 1118 prior to the next bag being delivered to the unit. The patient’s bradycardia worsened, leading to a Pulseless Electrical Activity (PEA) cardiac arrest. CPR was initiated and the code was managed by the code team. Ultimately CPR was stopped at the request of the family.

**Results:**

Pre-mixed Epinephrine infusion bags added to all adult ICU Omnicells. Pre-mixed Vasopressin infusion bottles added to all adult ICU Omnicells. Pre-mixed Norepinephrine infusion bags added to all Crash Carts throughout the hospital (pre-mixed norepinephrine infusion bags were already available in the ICU Omnicells). How To Mix signage was added to all Adult ICU area medication rooms or near the Omnicells.

**Conclusions:**

Due to the unfamiliarity in mixing an epinephrine infusion bag on the unit, the medication ran empty prior to the delivery of a replacement bag. This led to a hospital review and implementation of pre-mixed epinephrine being added to the Burn ICU Omnicell and other adult ICU Omnicell’s throughout the hospital. All together several improvements were made after reviewing this incident.

**Applicability of Research to Practice:**

This event led to a hospital change in the availability of pre-mixed vasopressors for the adult ICUs. More research could be done to compare the outcomes of critically ill patients on vasopressors mixed within the facility versus pre-mixed vasopressors. Additional research could be done comparing the number of adverse medication events with critically ill patients on vasopressors mixed within the facility versus pre-mixed vasopressors.

**Funding for the Study:**

N/A

